# Supplementary material for: Rapid Nuclear Exclusion of Hcm1 in Aging Saccharomyces cerevisiae Leads to Vacuolar Alkalization and Replicative Senescence
Source: G3 (Bethesda). 2018 Mar 8;8(5):1579–92. doi: 10.1534/g3.118.200161 (PMC5940150; doi:10.1534/g3.118.200161)
Supplement: Supplementary file 1 [file 1579FigureS1.pptx]

## Slide 1
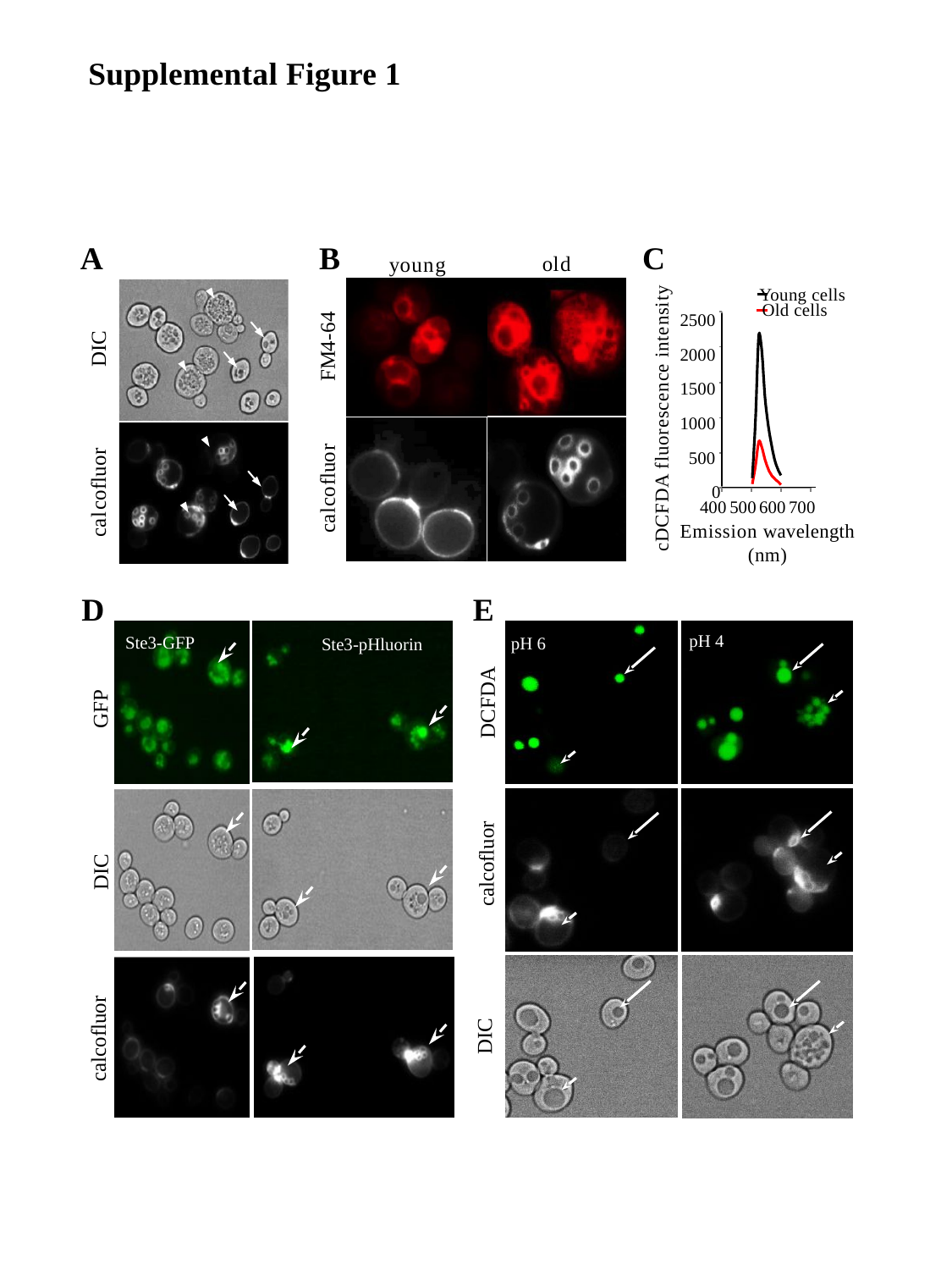

Supplemental Figure 1
A
B
C
old
young
FM4-64
calcofluor
Young cells
Old cells
2500
2000
1500
cDCFDA fluorescence intensity
1000
500
0
400
500
600
700
Emission wavelength (nm)
DIC
calcofluor
D
E
pH 4
pH 6
DCFDA
calcofluor
DIC
Ste3-GFP
Ste3-pHluorin
GFP
DIC
calcofluor
