# Supplementary material for: Rapid Nuclear Exclusion of Hcm1 in Aging Saccharomyces cerevisiae Leads to Vacuolar Alkalization and Replicative Senescence
Source: G3 (Bethesda). 2018 Mar 8;8(5):1579–92. doi: 10.1534/g3.118.200161 (PMC5940150; doi:10.1534/g3.118.200161)
Supplement: Supplementary file 2 [file 1579FigureS2.pptx]

## Slide 1
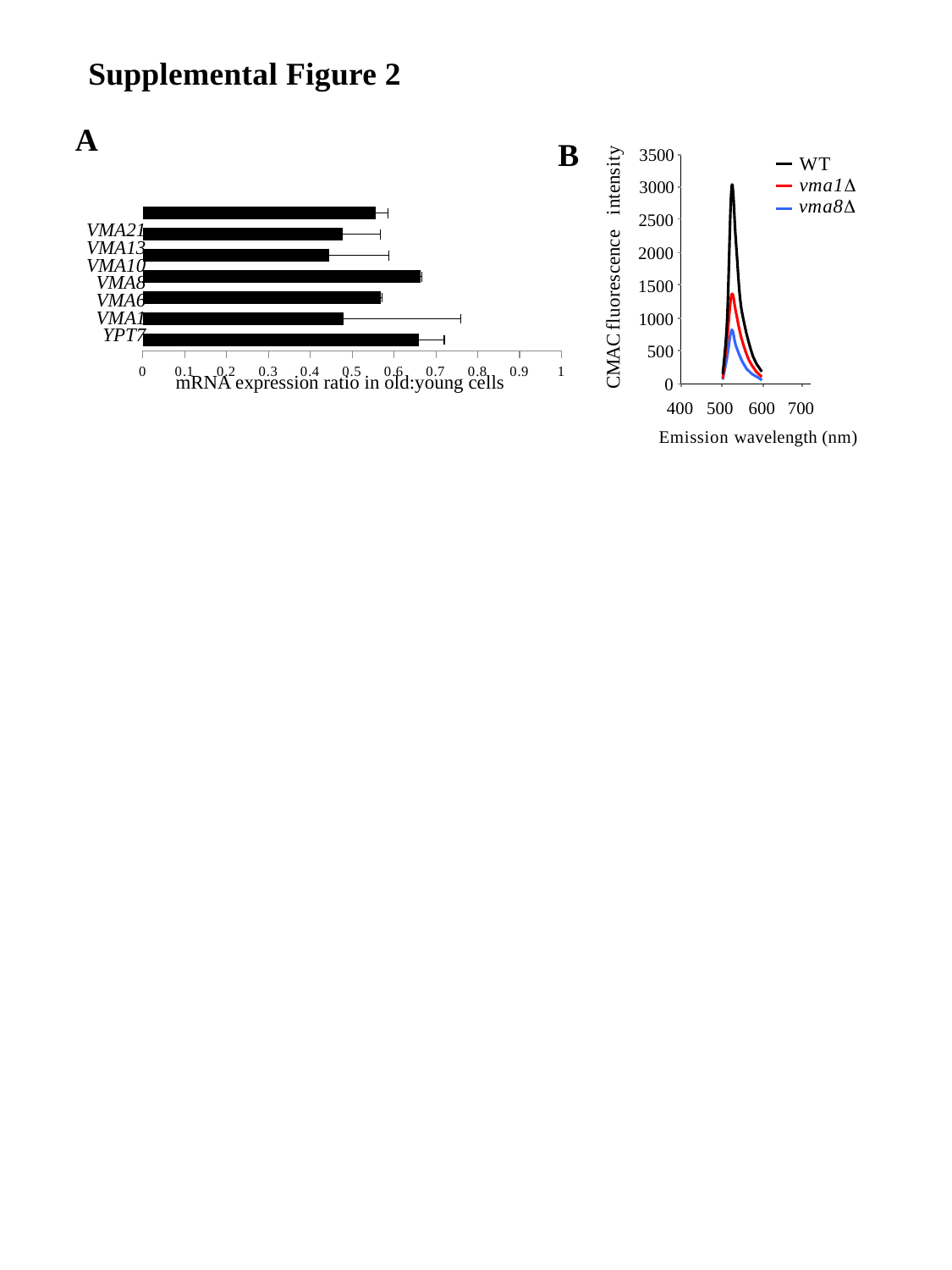

Supplemental Figure 2
CMAC fluorescence intensity
3500
WT
vma1D vma8D
3000
2500
2000
1500
1000
500
0
Emission wavelength (nm)
400
500
600
700
A
B
### Chart
| Category | |
|---|---|VMA21
VMA13
VMA10
VMA8
VMA6
VMA1
YPT7
mRNA expression ratio in old:young cells
