# Supplementary material for: Rapid Nuclear Exclusion of Hcm1 in Aging Saccharomyces cerevisiae Leads to Vacuolar Alkalization and Replicative Senescence
Source: G3 (Bethesda). 2018 Mar 8;8(5):1579–92. doi: 10.1534/g3.118.200161 (PMC5940150; doi:10.1534/g3.118.200161)
Supplement: Supplementary file 3 [file 1579FigureS3.pptx]

## Slide 1
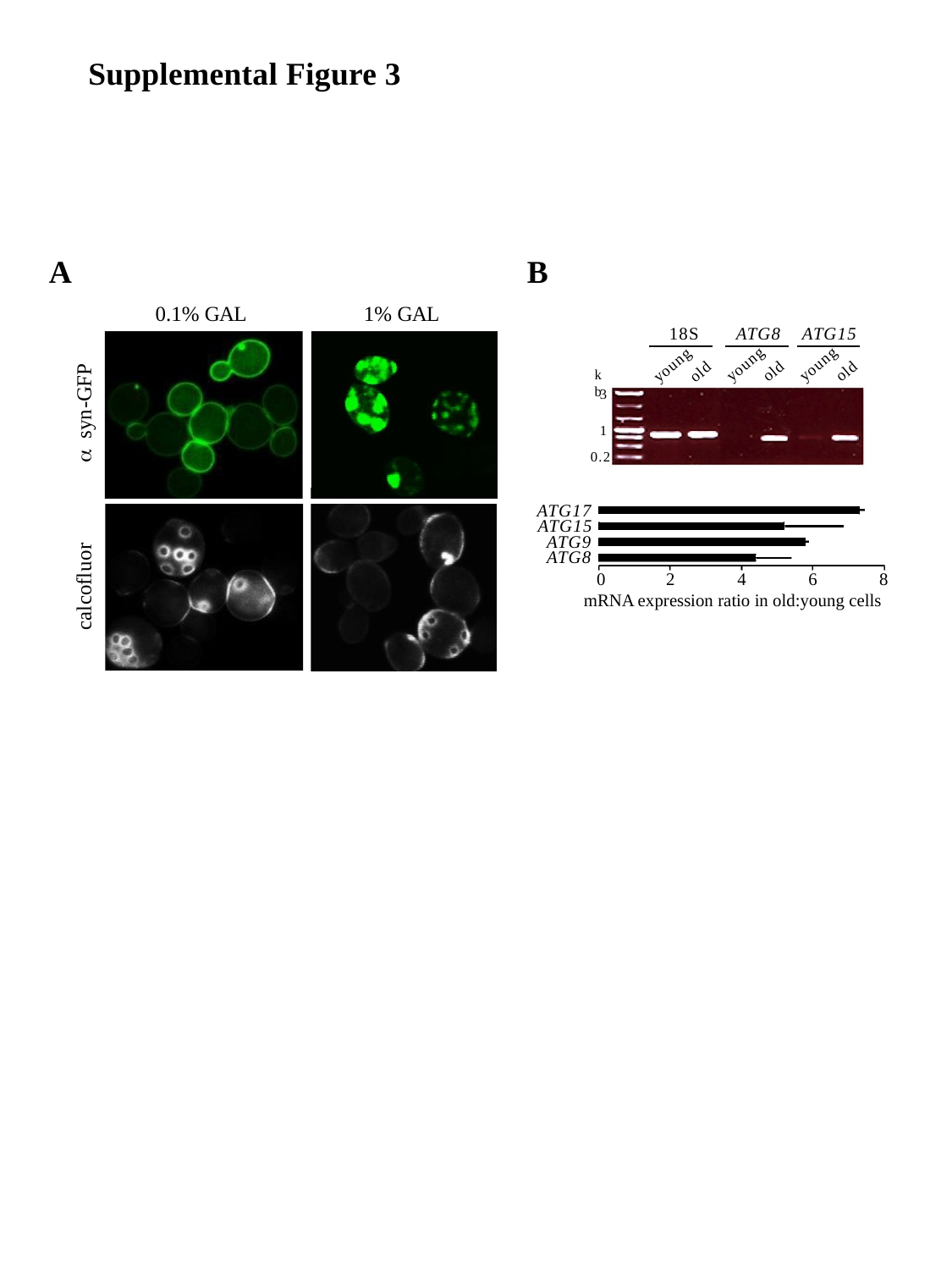

Supplemental Figure 3
A
B
0.1% GAL
1% GAL
a syn-GFP
calcofluor
18S
ATG8
ATG15
young
old
young
old
young
old
kb
3
1
0.2
ATG17
ATG15
ATG9
ATG8
0
2
4
6
8
mRNA expression ratio in old:young cells
