# Supplementary figures and images for: Rapid Nuclear Exclusion of Hcm1 in Aging Saccharomyces cerevisiae Leads to Vacuolar Alkalization and Replicative Senescence
Source: G3 (Bethesda). 2018 Mar 8;8(5):1579–92. doi: 10.1534/g3.118.200161 (PMC5940150; doi:10.1534/g3.118.200161)

## Slide 1
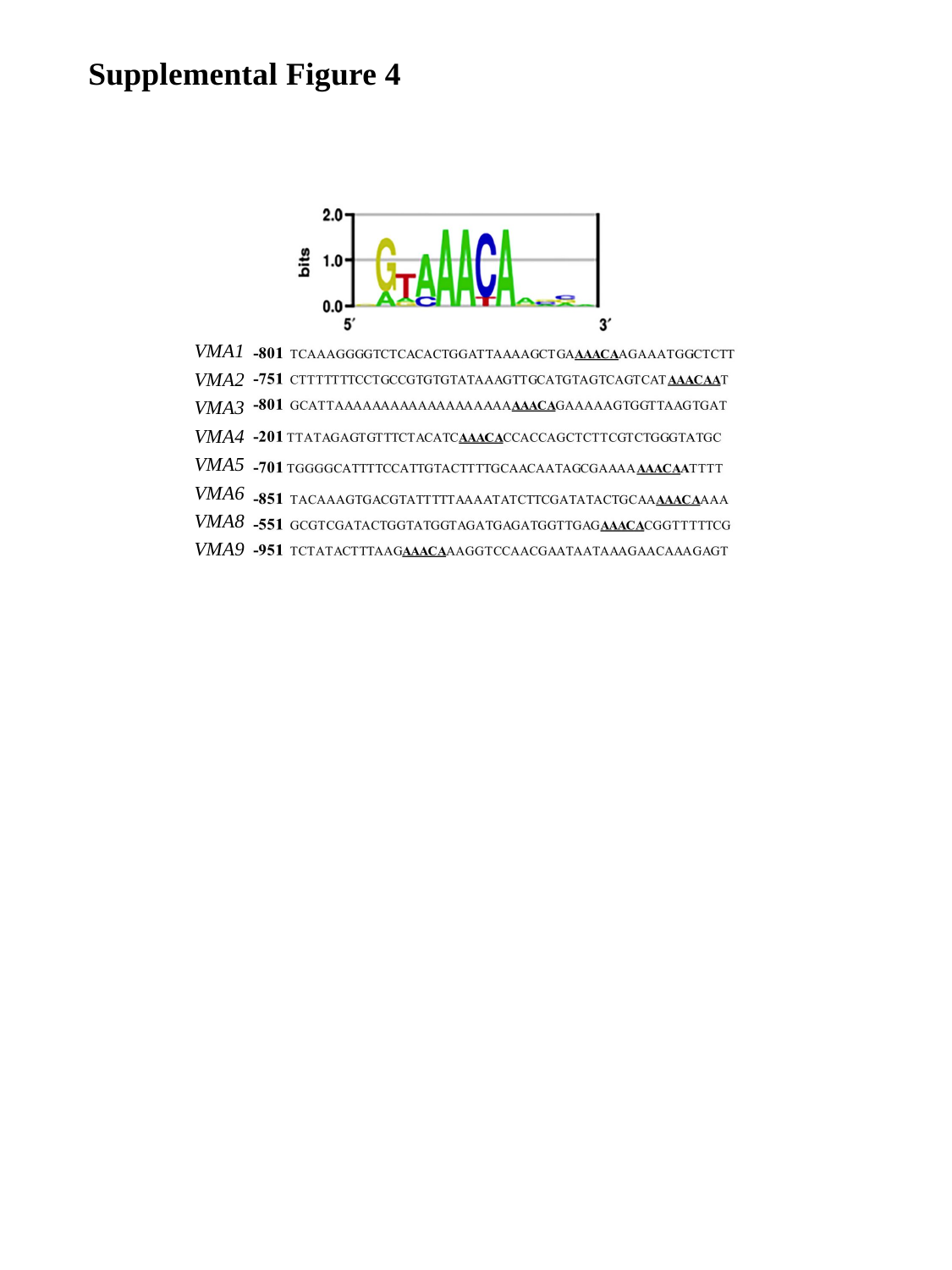

Supplemental Figure 4
VMA1
VMA2
VMA3
VMA4
VMA5
VMA6
VMA8
VMA9

Supplement: Supplementary file 4 [file 1579FigureS4.pptx]
