# Supplementary material for: Rapid Nuclear Exclusion of Hcm1 in Aging Saccharomyces cerevisiae Leads to Vacuolar Alkalization and Replicative Senescence
Source: G3 (Bethesda). 2018 Mar 8;8(5):1579–92. doi: 10.1534/g3.118.200161 (PMC5940150; doi:10.1534/g3.118.200161)
Supplement: Supplementary file 5 [file 1579FigureS5.pptx]

## Slide 1
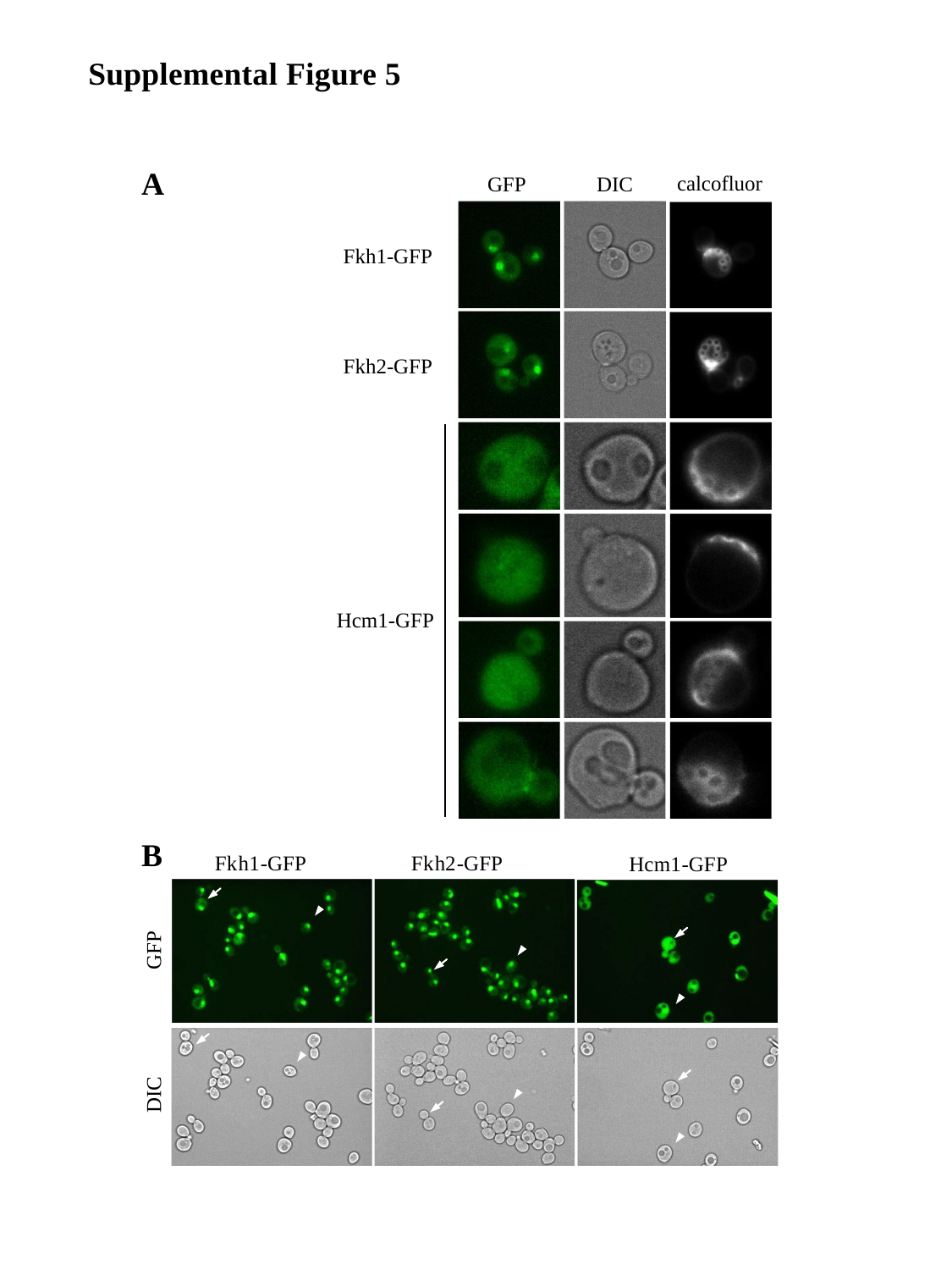

Supplemental Figure 5
A
Hcm1-GFP
Fkh1-GFP
Fkh2-GFP
GFP
DIC
calcofluor
B
Fkh1-GFP
Fkh2-GFP
Hcm1-GFP
GFP
DIC
