# Supplementary material for: Rapid Nuclear Exclusion of Hcm1 in Aging Saccharomyces cerevisiae Leads to Vacuolar Alkalization and Replicative Senescence
Source: G3 (Bethesda). 2018 Mar 8;8(5):1579–92. doi: 10.1534/g3.118.200161 (PMC5940150; doi:10.1534/g3.118.200161)
Supplement: Supplementary file 6 [file 1579FileS1.pdf]

Table S1. Statistical analysis for RTLS of vma1Δ mutants (Fig. 2D).

	vma1	*vma8*	*ypt7*	**WT**
T-test (compared with wt)	0.0032	9.64E-05	0.10	-
Mann-Whitney Test (compared to wt)	0.0403	1.75E-04	0.10	-
Significant difference from wt ($p < 0.05$)	Yes	Yes	No	-
Mean Lifespan (nmd*)	13.25	10.63	17.83	20.50
Maximal Lifespan (top 10%, nmd)	17.45	14.30	24.50	40.05

* nmd – number of mitotic divisions

Table S2. Cytoplasmic and nuclear GFP signal in cells with 1-2 bud scars.

N	Cytoplasmic Area (Pixels)	Total Cytoplasmic GFP Signal	Nuclear Area (Pixels)	Total Nuclear GFP Signal	Percent Nuclear GFP
1	1150	114971	63	9260	8.05
2	1432	140398	246	31934	22.75
3	1478	103989	174	19359	18.62
4	1223	135378	114	21234	15.69
5	1261	149667	100	19009	12.70
6	2445	306720	383	67458	21.99
7	1980	159408	199	26071	16.35
8	1855	189781	337	53669	28.28
9	2316	196770	339	45405	23.08
10	1894	144688	113	13751	9.50
11	2516	282001	384	59480	21.09
12	1018	181786	340	75220	41.38
13	2346	316780	299	66492	20.99
14	2305	248896	160	29970	12.04
15	2415	323967	299	66492	20.52
16	2588	293826	246	42684	14.53
17	2219	188074	78	12824	6.82
18	2358	238505	123	19057	7.99
19	1990	284520	163	37912	13.32
20	1450	133631	60	8891	6.65

Table S3. Cytoplasmic and nuclear GFP signal in cells with 3-4 bud scars.

N	Cytoplasmic Area (Pixels)	Total Cytoplasmic GFP Signal	Nuclear Area (Pixels)	Total Nuclear GFP Signal	Percent Nuclear GFP
1	2493	244374	267	32318	13.22
2	3380	379655	454	77361	20.38
3	2965	414534	381	69748	16.83
4	2829	248457	287	36334	14.62
5	2454	211792	172	17295	8.17
6	2512	192040	126	10861	5.66
7	2184	180562	72	7539	4.18
8	1669	124886	43	4193	3.36
9	2280	304644	146	26380	8.66
10	3473	305624	76	8262	2.70
11	3016	194065	149	13277	6.84
12	3502	340647	203	33624	9.87

Table S4. Cytoplasmic and nuclear GFP signal in cells with 5+ bud scars.

N	Cytoplasmic Area (Pixels)	Total Cytoplasmic GFP Signal	Nuclear Area (Pixels)	Total Nuclear GFP Signal	Percent Nuclear GFP
1	2084	274575	216	17014	6.20
2	2642	284234	97	10566	3.72
3	3618	328938	117	11870	3.61
4	3340	264942	186	20844	7.87
5	3011	318145	253	36211	11.38
6	2593	192110	126	10770	5.61
7	3755	289785	142	16313	5.63
8	2685	182808	128	10523	5.76
9	2704	289247	248	31692	10.96
10	3482	234077	100	9420	4.02
11	3827	454908	229	27401	6.02
12	3512	245496	159	13179	5.37
13	4406	362927	225	25825	7.12
14	3405	352309	100	12918	3.67
15	2998	168308	54	4395	2.61
16	2184	180562	72	7539	4.18
17	3563	279232	202	23035	8.25
18	2602	179379	128	10523	5.87
19	2812	163765	73	5511	3.37
20	2063	141996	193	17636	12.42

Table S5. Summary of Hcm1-GFP imaging statistics.

Group	Average % Nuclear GFP	SEM	P-Value Versus 0 - 2 BS Group	P-Value Versus 3 - 4 BS Group
0 - 2 BS	17.12	1.84	N/A	0.00246
3 - 4 BS	9.54	1.56	0.00246	N/A
5+ BS	6.18	0.61	0.00001	0.04
